# Comprehensive Review of Lipid Management in Chronic Kidney Disease and Hemodialysis Patients: Conventional Approaches, and Challenges for Cardiovascular Risk Reduction

**DOI:** 10.3390/jcm14020643

**Published:** 2025-01-20

**Authors:** Erica Abidor, Michel Achkar, Ibrahim Al Saidi, Tanvi Lather, Jennifer Jdaidani, Alaukika Agarwal, Suzanne El-Sayegh

**Affiliations:** 1 Department of Medicine, Northwell Health, Staten Island University Hospital, Staten Island, NY 10305, USA; eabidor@northwell.edu (E.A.); malachkar@northwell.edu (M.A.); ialsaidi@northwell.edu (I.A.S.); tlather@northwell.edu (T.L.); jjdaidani@northwell.edu (J.J.); aagarwal11@northwell.edu (A.A.); 2 Department of Medicine, Division of Nephrology, Northwell Health, Staten Island University Hospital, Staten Island, NY 10305, USA

**Keywords:** chronic kidney disease, hemodialysis, dyslipidemia, statins

## Abstract

**Background/Objectives**: Lipid disorders are very prevalent in patients with chronic kidney disease (CKD) and end-stage kidney disease (ESKD), leading to heightened cardiovascular risk. This review examines the effectiveness of lipid-lowering agents in these populations and explores gaps in the current research. The goal of this review is to assess the efficacy of lipid-lowering therapies in CKD and ESRD patients and identify future research needs. It aims to provide a clearer understanding of how these treatments impact cardiovascular risk in high-risk populations. **Methods**: We conducted a literature search in Embase, PubMed, Cochrane, and Google Scholar databases using keywords including but not limited to: chronic kidney diseases, dialysis, hemodialysis, dyslipidemia, statins, ezetimibe, and lipid-lowering drugs. Findings from included studies were synthetized to provide an overview of the current management of dyslipidemia in ESRD and HD. **Results**: Statins show mixed results in CKD and ESRD, with limited benefits in reducing cardiovascular events in dialysis patients. Agents like PCSK9 inhibitors show promising results but require further research, while non-statin therapies like fibrates and omega–3 fatty acids have limited evidence for use in this population. **Conclusions**: The review underscores the need for further research into lipid-lowering agents in CKD and ESRD patients, highlighting the need for tailored lipid management strategies in vulnerable patients with unique risk factors. More studies are needed to refine treatment strategies and assess the role of exercise and accurate risk calculators in managing cardiovascular outcomes.

## 1. Introduction

Chronic kidney disease patients and end-stage kidney disease patients face an elevated risk of cardiovascular disease due to the atherogenic nature of their blood profile associated with renal dysfunction. In 2021 more than 20 million deaths were attributed to cardiovascular diseases [1]. Considering that in individuals with stage 4 CKD, cardiovascular disease mortality can account for 50% of all deaths [2], as well as the rising incidence of dialysis patients, with the number of dialysis patients in the US doubling between 2000 and 2019 [3], identifying and closely monitoring dyslipidemia is of utmost importance, especially considering the high mortality rate post-cardiovascular events [4]. Various risk factors contribute to lipid disorders in HD patients, such as the increasing age of patients on dialysis, the higher prevalence of comorbidities such as diabetes and obesity in patients with ESKD compared to the general population, and uremia-related factors [5]. Unlike other populations with dyslipidemia, the dysregulation of several enzymes involved in lipid metabolism further exacerbates dyslipidemia in patients on HD [6]. Due to the many factors contributing to dyslipidemia in HD patients, individualized treatment is crucial for effective management.

Management of hyperlipidemia in CKD and end-stage kidney disease (ESKD) patients has traditionally involved statins. However, conflicting evidence arises from trials like the Die Deutsche Diabetes Dialyse Studie (4D), An Assessment of Survival and Cardiovascular Events (AURORA), and Study of Heart and Renal Protection (SHARP), in which atorvastatin, rosuvastatin, and simvastatin plus ezetimibe showed varying impacts on lipid levels but failed to consistently improve cardiovascular outcomes in dialysis patients [7,8,9]. Alternative lipid-lowering agents, such as fibrates, bile acid sequestrants, and omega–3 fatty acids, have limited and sometimes conflicting evidence in CKD and ESKD patients.

The Kidney Disease Improving Global Outcomes (KDIGO) guidelines published in 2013, provide a comprehensive approach to lipid management in CKD. Since then, newer but less comprehensive guidelines have been released by the American Heart Association (AHA) in 2018 and the European Society of Cardiology (ESC) in 2019, but recommendations remain non-specific [10,11].

This review examines lipid disorders in CKD patients on hemodialysis, evaluating treatment strategies, emerging therapies, and the role of exercise while highlighting studies, clinical outcomes, and future directions to optimize care and quality of life for hemodialysis patients.

## 2. Methods

A comprehensive literature review was conducted in Embase, PubMed, Cochrane, and Google Scholar databases for articles addressing or reviewing lipid management in individuals with CKD and ESKD. We used the MeSH term and free text for our search strategy. The following keywords were used: chronic kidney diseases, dialysis, hemodialysis, dyslipidemia, statins, ezetimibe, lipid-lowering drugs, LDL, Cholesterol, triglycerides, PCSK-9 inhibitors, and Omega–3 fatty acids. Studies on the pediatric population and studies reported in a language other than English were excluded from this review. Findings from included studies were synthetized narratively to provide an overview of the current management of dyslipidemia in ESKD and HD.

Since this is a narrative review, certain limitations must be addressed. First, since narrative reviews do not systematically include all studies on the subject, they might be more susceptible to publication or selection bias. In addition, the absence of quantitative synthesis limits our ability to draw firm conclusions.

This study is a literature review and does not involve the collection of primary data or the direct involvement of human participants; therefore, ethical approval and informed consent are not applicable.

## 3. Prevalence of Lipid Disorders in Hemodialysis Patients

The dyslipidemia associated with CKD is the result of an intricate group of interactions involving genetic, environmental, and kidney-specific factors [6]. In a study that was in line with the Kidney Disease Outcomes Quality Initiative (KDOQI), 21,893 dialysis patients had their lipid levels measured immediately prior to dialysis. Dyslipidemia was determined to be 82% prevalent in these patients, and over half of the patients included had hypertriglyceridemia, elevated very-low-density lipoprotein cholesterol, and decreased high-density lipoprotein (HDL) [12]. Similar findings were found in research by Hassan et al., which found a prevalence of dyslipidemia of 72% in a sample of ESKD patients on HD with low HDL being the most frequently observed lipid abnormality [13].

It has been noted that individuals who were on HD had a higher prevalence of dyslipidemia compared to the general population, where the prevalence of dyslipidemia ranges from as low as 9% in an Indonesian study to 53% in a study carried out in the United States [14,15].

This higher prevalence can be explained by specific derangement to several enzymes that play a major role in lipid metabolism such as lipoprotein lipase and lecithin–cholesterol acyltransferase [16,17], in addition to several other risk factors that will be explained in the next section of our review. Cardiovascular disease has been determined to be the most important cause of death amongst patients with impaired kidney function, with patients with CKD having a high burden of cardiovascular disease (CVD) [18]. As such, it is very important to identify and monitor dyslipidemia closely in CKD patients on HD due to its high prevalence and their increased risk of adverse cardiovascular events.

## 4. Risk Factors for the Development of Lipid Disorders in Hemodialysis Patients

The factors contributing to the high prevalence of lipid disorders in hemodialysis patients are wide-ranging. They include increased age, male gender, and associated co-morbidities such as diabetes, hypertension, and obesity, along with risk factors associated with uremia, which include anemia, hyperhomocysteinemia, oxidative stressors, hyperparathyroidism associated with mineral bone disease, and chronic inflammation [5]. Increased oxidative stress in patients with CKD and ESKD may result from elevated levels of uremic toxins and free radicals, combined with mitochondrial dysfunction that impairs the ability to scavenge free radicals or degrade proteins damaged by oxidation [19]. Uremic toxins such as indoxyl sulfate, indole-3 acetic acid, p-cresyl sulfate, trimethylamine N-oxide, and phenylacetylglutamine, derived from dietary protein metabolism by gut microbiota, are linked to cardiovascular disease in CKD [20]. Indoxyl sulfate induces oxidative stress, inhibits nitric oxide production, and impairs vascular endothelial cell function, contributing to atherosclerosis in CKD through inflammation and endothelial dysfunction [20]. Furthermore, patients with ESKD have been shown to be more at risk of oxidative stress compared to patients with CKD due to the filtration of antioxidant molecules during dialysis and the activation of leukocytes by the dialysis membrane [21]. Individuals with CKD and ESKD also differ significantly in their dietary recommendations. Patients with CKD are typically advised to follow a low-protein diet to slow disease progression, whereas patients with ESKD undergoing dialysis are often encouraged to consume a high-protein diet to compensate for protein loss during dialysis [22]. These differences in dietary protein intake can influence lipid profiles, as high-protein diets have been associated with increased HDL cholesterol levels and lower triglyceride levels [23]. According to current research, dyslipidemia in HD patients may result from a decreased ability to convert free fatty acids through β-oxidation. This can be due to elevated levels of lipase inhibitors, such as apolipoprotein C-III, causing accumulated fatty acids to be converted to complex lipids, such as triglyceride-rich proteins [6].

Apolipoprotein A1, the key protein component of HDL, and apolipoprotein B, the major protein component of VLDL and LDL, are closely linked to chronic kidney disease (CKD). Lower levels of apolipoprotein A1 and higher levels of apolipoprotein B have been identified as significant markers associated with CKD [24].

It has also been shown that the increased concentrations of VLDLs and chylomicrons are due to the delayed breakdown and increased production of triglyceride-rich proteins in patients with impaired kidney function [5]. Patients with CKD exhibit reduced activity of hepatic triglyceride lipase and peripheral lipoprotein lipase leading to elevated levels of triglycerides in patients with CKD [5].

Various hormone imbalances in this population are linked to altered lipid profiles, contributing to increased cardiovascular risks in CKD. Secondary hyperparathyroidism, commonly seen in patients undergoing HD, is believed to contribute to the decreased breakdown of triglyceride-rich proteins [25,26]. Similarly, insulin resistance, prevalent in CKD patients, is linked to increased hepatic VLDL overproduction [16]. Testosterone deficiency, prevalent in 40% to 60% of male dialysis patients [27], is associated with increased fat mass, reduced insulin sensitivity, impaired glucose tolerance, elevated triglycerides and LDL levels, and decreased HDL cholesterol. These metabolic disturbances, especially low HDL cholesterol, significantly contribute to the progression of atherosclerosis [28,29].

It is noteworthy that individuals with CKD not only have lower HDL levels but also have impaired HDL function. HDL usually serves as a transporter of cholesterol from peripheral sites to the liver, lowering the amount of cholesterol present peripherally. In CKD patients, HDL does lose some of its activity due to modifications in lipoprotein metabolism. As a result, HDL loses its ability to reduce inflammation and instead becomes pro-atherogenic [12]. This dysfunction of HDL in CKD highlights the need for interventions that can positively influence lipid metabolism and cardiovascular risk factors in this population.

Sevelamer, a widely used phosphate binder for CKD and ESKD patients, has been extensively studied for its potential effects beyond phosphate control. In one of the most recent trials on sevelamer and lipid levels, sevelamer reduced total cholesterol by 17% compared to 3% with a placebo and lowered LDL cholesterol by 33.5% compared to 7.6% with a placebo [30]. A meta-analysis conducted by Basutkar et al. confirmed findings from previous studies consistent with sevelamer’s ability to increase HDL levels and decrease total cholesterol [31].

## 5. The Role of Physical Activity in Patients with CKD and ESRD

In its 2019 guidelines, the American College of Cardiology stressed the importance of physical activity in reducing the risk of atherosclerotic cardiovascular diseases [10]. Physical activity is limited in dialysis patients when compared to the elderly population not on dialysis, which can be attributed to the sedentary lifestyle on dialysis days and post-dialysis fatigue syndrome, both of which contribute to the development of sarcopenia [32,33,34,35,36]. Dialysis initiation has been associated with a decline in functional status, independent of demographic variables or pre-dialysis functional trajectory, further exacerbating the sedentary behavior observed in this population [37], with patients on peritoneal dialysis suffering from higher rates of fatigue and greater functional decline than patients on HD [38,39]. In addition, the presence of anemia in patients with CKD has been associated with a decreased prevalence of high-intensity physical activity compared to patients with CKD but with no anemia [40]. While advancements in dialysis have improved patient life expectancy, they have also led to a higher likelihood of developing adverse outcomes related to prolonged dialysis, such as malnutrition or dialysis-related amyloidosis [41,42]. As a result, the concept of renal rehabilitation, introduced by the Japanese Society of Renal Rehabilitation (JSRR) in 2011, emerged as a response to this complex dynamic between extended life expectancy and heightened medical issues among dialysis patients. Renal rehabilitation encompasses a comprehensive program aimed at alleviating the physical and mental effects of kidney disease, ultimately enhancing life expectancy and improving psychological and occupational circumstances [43]. It has also been shown that regular exercise on inter-dialysis days increases the 60 min sit-to-stand score, which is a surrogate for lower body strength and endurance [44]. The KDOQI guidelines even recommended exercise for patients on dialysis but advised them to skip exercise on dialysis days to avoid the risks of postural hypotension or dizziness [45]. The beneficial effect of exercise on patients requiring HD was also highlighted in the study by Matsuzawa et al., which showed that patients on HD who had more than 50 min of physical activity per day, such as gentle walking or running, were found to decrease mortality by 22% [46]. Undergoing pre-dialysis yoga exercises was found to decrease LDL cholesterol levels by 11.32% and triglyceride levels by 6.26% in individuals with ESKD on HD [47].

Currently, no clinical guidelines or professional societies provide explicit recommendations for exercise in patients undergoing dialysis [48]. However, the majority of published studies suggest engaging in exercise two to three times per week, primarily on interdialytic days [48]. The recommended duration of exercise for dialysis patients varies, ranging from 20 min per session to as long as 90 min [49]. Regarding the type of exercise, the literature recommends multiple activities, such as walking, swimming, and stationary biking [50].

Given the multifaceted nature of managing patients on hemodialysis, healthcare providers must prioritize the integration of physical activity into patient care to optimize outcomes and enhance their quality of life.

## 6. Management of Hyperlipidemia in CKD and ESRD Patients

Many randomized trials have proven beneficial effects of statins in cardiovascular diseases in patients with impaired renal function [51,52,53]. The Assessment of Lescol in Renal Transplantation (ALERT) study, along with a complex extension study of ALERT, looked at the effect of Fluvastatin on cardiovascular outcomes and found a substantial decrease in non-lethal myocardial infarction and cardiac death in kidney transplant recipient patients [54,55].

Despite progress, dedicated research is essential for refining lipid-lowering agents for dialysis. Statin therapy’s cost-effectiveness is established in the general population, but the evidence is limited in patients with ESKD on dialysis.

Three randomized controlled trials The Die Deutsche Diabetes Dialyse Studie (4D), An Assessment of Survival and Cardiovascular Events (AURORA), and Study of Heart and Renal Protection (SHARP) looked at the role of statins in lipid-lowering and cardiovascular outcomes in dialysis patients [7,8,9]. The 4D, AURORA, and SHARP trials did not show any benefit of statin therapy in patients on dialysis [7,8,9].

Statin administration in patients with CKD should be monitored closely. In individuals using statins, chronic kidney disease (CKD) is one of the risk factors for the development of myopathy and rhabdomyolysis, which might worsen renal function [56,57]. Although there are currently no studies or trials evaluating the impact of CKD on liver damage in statin users, it has been demonstrated that patients with CKD do not have an increased incidence of abnormal liver function [58].

### 6.1. Statins and Ezetemib

#### 6.1.1. Atorvastatin

In the 4D study, atorvastatin 20 mg or a placebo was randomly allocated to 1255 diabetic individuals who had started hemodialysis less than two years before the study’s start date, and 29% of whom had cardiovascular disease. The group that received Atorvastatin showed a significant reduction in LDL cholesterol levels in 4 weeks. However, atorvastatin did not lead to a significant reduction in the combined outcome of major cardiovascular events compared to placebo over a median follow-up of 4 years. Atorvastatin did lower the overall rate of cardiac events compared to the placebo [8].

The lack of clear benefit in diabetic patients receiving statins may be explained by other pathophysiological mechanisms contributing to increased cardiac mortality, such as cardiac micro-vessel disease or left ventricular hypertrophy [8]. Additionally, the 20 mg daily dose of statin used in this study, considered a moderate-intensity dose, may not have provided sufficient lipid control compared to high-intensity statins, and therefore might not have had the desired effect on cardiac mortality [8].

#### 6.1.2. Rosuvastatin

In the AURORA study, 2776 patients who had undergone hemodialysis for a minimum of 3 months and had not previously received statin therapy were assigned to receive either 10 mg of rosuvastatin daily or a placebo. Approximately 40% of the participants had a history of cardiovascular disease, and 10% had a previous myocardial infarction. Unlike the 4D study, 74% of AURORA participants were diabetic, with recruitment limited to patients aged between 50 and 80 years old, while 4D included those over 18 years old. Although rosuvastatin significantly lowered LDL cholesterol and C-reactive protein levels by 43%, there was no significant reduction in major cardiac events compared to placebo after a follow-up period of 3 years [7].

These findings are intriguing, as the decrease in LDL concentration did not correlate with a reduction in mortality. The AURORA study, which recruited patients aged 50 to 80 years, may have seen limited statin benefits compared to a younger population on statins [7]. Additionally, despite a reduction in inflammatory markers such as C-reactive protein, mortality did not differ between the two groups, suggesting other factors may contribute to inflammation in elderly patients with ESKD [7].

#### 6.1.3. Simvastatin and Ezetimibe

The SHARP trial, a double-blind, randomized trial, examined the effect of simvastatin combined with ezetimibe for primary prevention of atherosclerotic vascular events in 9270 patients with CKD and ESKD [9]. Among them, 6247 patients had elevated serum creatinine levels but did not require dialysis with more than 60% of them having CKD stage 4–5, while 3023 patients were on dialysis. Participants were first assigned to receive either simvastatin plus ezetimibe, simvastatin alone, or a placebo. After one year, those originally on simvastatin monotherapy were randomly switched to either combination therapy with simvastatin plus ezetimibe or a placebo. The group receiving combination therapy showed a significant reduction in LDL cholesterol levels compared to the placebo. The simvastatin plus ezetimibe group showed a significant decrease in major atherosclerotic events compared to placebo after an average follow-up of 4.9 years [9].

Despite patients with CKD and ESKD showing improved lipid profiles, better cardiovascular benefits such as decreased rate of on-hemorrhagic stroke, arterial revascularization procedures, and improved mortality outcomes were more evident in the CKD group than in the ESKD group [9]. The findings from the SHARP study may encourage more aggressive lipid control, potentially using two agents, to achieve optimal outcomes. Other studies have also demonstrated the benefits of combination therapy with ezetimibe. Adding ezetimibe to a moderate-intensity statin has been shown to reduce outcomes such as myocardial infarction and stroke by 15% [59].

A summary of the studies mentioned above is presented in Table 1:

### 6.2. Other Lipid Lowering Agents

#### 6.2.1. Fibrates

Although treatment with fibrates is not recommended by any major medical society in patients with CKD, new and ongoing research suggests that this drug might convey benefits in patients with CKD. Goto et al. showed a lower risk of major cardiovascular events in patients with CKD treated with fibrates [60]. Similar results were also seen in a study by Yen et al. in which patients with stage 3 CKD who were taking fibrates experienced fewer cardiovascular events than those who were not [61]. Fibrates are usually indicated in patients with CKD with a triglyceride level of 500 mg/dL and above [62]. Fibrates require dose titration in individuals with decreased renal function and should be avoided in patients undergoing dialysis.

#### 6.2.2. Bile Acid Sequestrants

Bile acid sequestrants, such as cholestyramine, work by binding to the bile salts in the intestinal tract and decreasing intestinal absorption of cholesterol, which leads to a reduction in the blood LDL levels; however, they can increase circulating triglyceride levels. This limits their use in patients with hypertriglyceridemia, including CKD and ESKD patients [63,64,65]. Bile acid sequestrants are thought to be well tolerated in patients with CKD or on HD due to the lack of systemic absorption [66]. The use of bile acid sequestrants in this particular population was discouraged due to the lack of research examining their safety in patients with reduced kidney function and the lack of evidence of cardiovascular benefit.

#### 6.2.3. Omega–3 Fatty Acids

Omega–3 fatty acids act by mainly lowering blood triglyceride levels. Research on the cardiovascular benefits of omega–3 fatty acid administration in the general population has been inconsistent, with some research revealing benefits while others have not [67,68].

Regarding the lipid-lowering effects of omega–3 fatty acids, a meta-analysis carried out by Chi et al. demonstrated that omega–3 supplementation in patients on dialysis resulted in a reduction in serum triglycerides and total cholesterol levels [69]. Another meta-analysis by Zhu et al. showed similar findings regarding reduction in cholesterol and triglyceride levels [70].

Despite conflicting evidence on cardiovascular mortality in the general population, increased omega–3 polyunsaturated fat intake was associated with a decreased cardiovascular mortality risk in patients on hemodialysis [71].

#### 6.2.4. Proprotein Convertase Subtilisin/Kexin Type 9 (PCSK9) Inhibitors

PCSK9 inhibitors are a relatively new class of lipid-lowering medications. Trials like ODYSSEY, PROFICIO, FOURIER, SPIRE, ORION-1, 2, and 3 studied the lipid-lowering effect of PCSK9 inhibitors in the general population and even CKD patients, but none of the trials included ESKD patients on dialysis [72,73,74,75,76,77,78,79,80,81]. According to an analysis that included CKD patients from the FOURIER research, evolocumab consistently reduced LDL-C in all CKD groups [82]. Myocardial infarction and cardiovascular death were also observed to be reduced in patients on evolocumab; with patients with CKD stage 3 having more risk reduction than those with stage 2 [82].

East et al. evaluated the safety and effectiveness of PCSK inhibitors in patients with end-stage kidney disease (ESKD). They found that alirocumab was equally effective in patients receiving maintenance dialysis and those not receiving dialysis, with no unexpected side effects [83].

### 6.3. Novel Lipid Lowering Agents

Numerous innovative therapies are under development for managing dyslipidemias and their related risks, which come with high production costs. Presently, there is limited evidence supporting the utilization of these new lipid-lowering agents in individuals with chronic kidney disease (CKD) or end-stage renal disease (ESKD). However, there is a clear need for additional research studies to further investigate the efficacy of these treatments in such patient populations. Cautious adoption of newer therapies is crucial to ensure meaningful outcomes without compromising safety.

#### 6.3.1. Cholesteryl Ester Transfer Protein (CETP) Inhibitors

Torcetrapib was the first CETP that underwent clinical trials. It had a lipid-lowering effect but led to an increase in blood pressure, aldosterone, and endothelin 1 levels, which led to an increased risk of cardiovascular events and eventual withdrawal from the market [84,85,86]. Evacetrapib and Dalcetrapib had a similar effect, and their development was stopped [72,87,88,89].

The phase III Randomized Evaluation of the Effects of Anacetrapib through Lipid-Modification (REVEAL) trial studied Anacetrapib in individuals with known atherovascular disease and showed a decrease in major coronary events, but patients with CKD (Cr > 2.3 mg/dL) and ESKD were not included in this study [90,91].

#### 6.3.2. ApoCIII Antisense Oligonucleotide

Volanesorsen demonstrated the ability to reduce levels of triglycerides and lower the occurrence of pancreatitis in individuals with familial chylomicronemia syndrome [92,93]. Ongoing research is being conducted to further investigate the effects of volanesorsen in patients with hypertriglyceridemia and other populations [93].

#### 6.3.3. Angiopoietin-like Protein 3 (ANGPTL3) Inhibitors

Evinacumab has been shown to decrease triglyceride and LDL cholesterol in healthy individuals [94,95]. No data are available on outcomes in patients with CKD or ESKD on dialysis.

## 7. Current Guidelines for the Use of Lipid-Lowering Agents in CKD and ESRD

The 2013 KDIGO guidelines for lipid treatment of CKD continue to be the most thorough resource for recommending cholesterol-lowering medications in patients with CKD and ESKD [96]. The American Heart Association (AHA) and the European Society of Cardiology (ESC) released updated guidelines on the management of dyslipidemia in CKD and ESKD. The KDIGO guidelines emphasize individualized treatment without strict LDL monitoring, aiming to reduce the treatment burden on patients with CKD while focusing on overall cardiovascular risk management [96]. The AHA guidelines specify the intensity of lipid-lowering therapy and recommend combination treatment in high-risk populations, suggesting a more aggressive approach for lipid management and reduction in cardiovascular disease risk [10]. The ESC does not include atherosclerotic cardiovascular disease (ASCVD) risk calculation in its algorithm for prescribing statins in patients with CKD [11].

Since the ASCVD risk calculator does not account for characteristics related to declining kidney function, it tends to underestimate the true risk in patients with CKD, despite being frequently utilized for decisions on the initiation of statins in the general population [97]. There is a need for clearer connections between the guidelines for statin use in CKD patients. The KDIGO, AHA, and ESC guidelines emphasize the importance of statins for cardiovascular risk reduction in CKD patients, but their sometimes divergent recommendations for different CKD stages make their applicability unclear. Harmonizing these approaches could provide more consistent guidance for statin therapy in CKD patients. Vallejo-Vaz et al. compared the ESC and AHA guidelines to assess the extent of absolute risk reduction. They found that implementing the ESC guidelines resulted in a 2% greater risk reduction in cardiovascular events compared to the AHA guidelines [98].

Regarding treatment objectives, the KDIGO guidelines do not recommend any lipid profile measurements or testing after the initiation of statins [96]. The ESC guidelines, on the other hand, recommend monitoring LDL levels after the initiation of statins, with a target LDL reduction of more than 50%, aiming for an LDL level of less than 70 mg/dL in patients with CKD stage 3, and less than 55 mg/dL in patients with CKD stage 4 and 5 [11]. Statins should be started in patients with LDL levels of 190 mg/dL or more, according to AHA guidelines, and in those with LDL levels between 70 and 189 mg/dL if the ASCVD risk score is greater than 7.5% [10]. Annual screening of lipid profiles in patients with CKD is considered a reasonable approach for monitoring and managing lipid levels [99].

**Figure 1:**
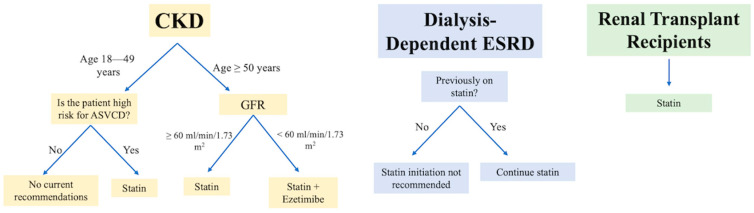
KDIGO clinical practice guideline for lipid management in CKD.

**Figure 2:**
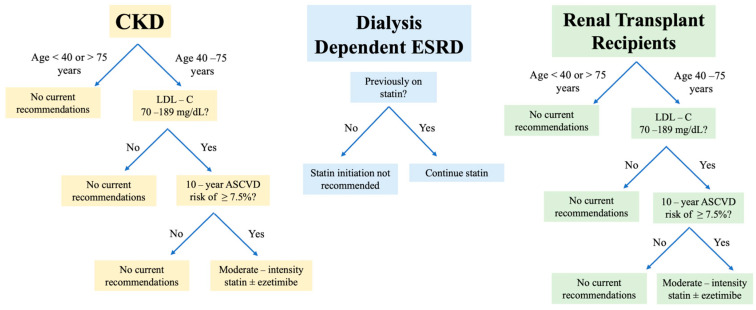
AHA/ACC clinical practice guidelines for lipid management in CKD.

**Figure 3:**
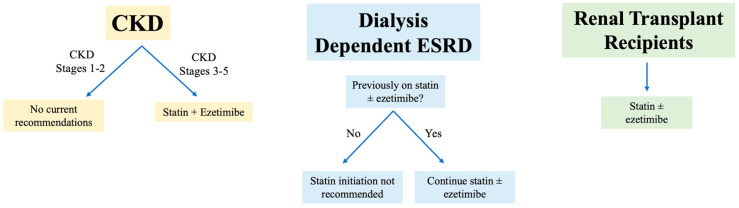
ESC clinical practice guidelines for lipid management in CKD.

Figure 1, Figure 2 and Figure 3 illustrate the current guidelines for the management of hyperlipidemia in patients with CKD and ESKD.

Regarding the triglyceride-lowering treatment, adults with CKD and hypertriglyceridemia are advised to implement therapeutic lifestyle changes [62]. While no specific target exists for triglyceride levels in patients with CKD or ESKD, the general population’s target is to reduce triglyceride levels by 35% to 50% [62]. No current clinical practice guideline recommends the initiation of fibrates in patients with CKD or HD.

## 8. The Outcomes of Long-Term Dialysis Patients on Lipid-Lowering Medications

Multiple studies have explored the efficacy of statins for a reduction in cardiovascular mortality. However, despite many attempts to deduce a specific set of risk factors that would justify using statins for reduction in CVD mortality in HD patients, no clear recommendations can be made. This is best reflected when reviewing the KDIGO 2013 guidelines, where starting a statin or ezetimibe combination in HD patients depends on whether the patient was already on lipid-lowering therapy before HD was initiated [100]. Reviewing the current literature and understanding the physiological changes induced by HD highlights why it is still a challenge to provide a more unified approach for the use of lipid-lowering therapy in HD patients. It was shown that malnutrition–inflammation markers which are higher in HD patients were associated with lower survival of dialysis patients after ACS, whereas LDL cholesterol levels were not associated [101]. Some studies suggested that decreasing renal parameters results in a distinctive cardiovascular phenotype associated with a high proportion of cardiovascular mortality caused by heart failure and heart rhythm abnormalities, possibly justifying the reduced benefit of statins on overall CVD mortality [100].

Multiple meta-analyses were conducted to further explore this topic. Herrington et al. revealed that as GFR declines, statin therapy results in smaller relative risk reductions for major cardiac events and strokes, even after accounting for the smaller decreases in LDL levels [102].

Studies have indicated that in dialysis patients, the relative risk reduction is notably smaller in advanced stages of CKD, and initiating statin therapy is generally not recommended for most patients on long-term hemodialysis [96]. A post hoc analysis of a large clinical trial showed that statins had beneficial effects in renal transplant recipients and patients in the initial stages of CKD, although the evidence is uncertain for those in the more advanced stages of CKD [103]. Patients with kidney transplants exhibit a unique cardiovascular profile influenced by factors such as obesity, hypertension, episodes of acute kidney rejection, and the effects of immunosuppressants and steroids commonly used in post-transplant care [104]. The benefits of statins in this population extend beyond lowering LDL levels, as they also contribute to improved patient and graft survivability by reducing cardiovascular events [105].

A study on the Korean population illustrated that statins are useful in ESKD when started after the first acute coronary event. More than half of the dialysis patients in the study were on statins following their first cardiovascular event, with the majority receiving moderate or high-intensity statin therapy [106]. The study concluded that the positive outcome of statins on all-cause mortality is more noticeable in high-risk dialysis patients, such as patients aged 65 years and above or those with diabetes [106]. In a study involving 14,298 patients transitioning to dialysis, continuing statin therapy was linked to a 28% reduction in the risk of 12-month all-cause mortality and an 18% reduction in cardiovascular mortality, compared to discontinuing statin treatment [107]. However, the benefit diminished as the duration of dialysis increased [107]. According to those results, the continuation of statins in patients with pre-existing cardiovascular disease after transitioning to hemodialysis has been shown to provide significant cardiovascular benefits.

Elderly patients on dialysis present additional challenges such as polypharmacy, age-related debility, and greater susceptibility to side effects [108]. These factors can limit the use of statins in this population, even when multiple cardiovascular risk factors are present.

There have been no recent trials assessing the benefit of statins for primary prevention of cardiovascular events in diabetic patients with ESKD undergoing hemodialysis. A study published in 2005 found that atorvastatin had no statistically significant effect on cardiovascular outcomes in diabetic patients undergoing dialysis [8]. In contrast, a study conducted in 2011 showed that rosuvastatin reduced the risk of both fatal and nonfatal cardiac events in diabetic patients with ESKD on dialysis [109]. These contradictory findings underscore the need for an individualized approach to statin initiation in this population, particularly given the high heterogeneity of patients on dialysis.

## 9. Future Directions

More research should concentrate on approving the use of the novel anti-lipidemic medications in this specific population, as the majority of the studies in this area did not include or even exclude dialysis patients or people with chronic renal failure.

An analysis of the ORION-1 and ORION-7 studies investigating inclisiran, a small interfering RNA targeting PCSK9, revealed that the safety profile and the pharmacodynamic effects of the drug were comparable in patients with mild, moderate, and severe impaired kidney function compared to patients with normal kidney function [110]. Patients on dialysis were excluded from the study [110]. Even though this finding opens the door for inclisiran use in this population, more research is required to assess for less frequent adverse reactions, especially in patients on dialysis.

The widespread use of PCSK9 inhibitors faces multiple barriers, primarily due to their elevated cost and the need for insurance authorizations [111], even after recent price reductions [112]. Studies have shown that PCSK9 inhibitors are not cost-effective for patients in China [113]. Conversely, a study conducted in Spain demonstrated their cost-utility, highlighting improvements in self-care and daily activities among patients treated with PCSK9 inhibitors [114].

Bempedoic acid was also found to be highly effective at reducing LDL-C levels in patients with CKD stage 2 and CKD stage 3a and 3b with no increased rate of adverse events. More large-scale trials are also needed before this agent can make its way to treatment guidelines for lipid-lowering agents in patients with CKD [115]. This drug has not been adequately studied in patients with a GFR of less than 30 mL/min/1.73 m^2^ or those on dialysis, restricting its applicability in this population [116].

None of the medications used to treat hypercholesterolemia in CKD patients have been demonstrated to slow CKD progression. Several drugs are currently being investigated such as cyclodextrins that are capable of improving renal lipid metabolism and delay CKD progression [117].

Regarding ASCVD risk calculation in patients with CKD, the ASCVD risk estimator tends to underestimate the risk of cardiovascular disease in people with CKD. Recently, the PREVENT calculator—a new cardiovascular event risk calculator—was suggested, and one of its parameters is glomerular filtration rate [118]. Additionally, the PREVENT calculator can calculate the risk of atherosclerotic cardiovascular disease for up to 30 years, while the ASCVD risk calculator can only calculate the risk for 10 years [118]. It may be possible to more accurately determine whether individuals with compromised kidney function require cholesterol-lowering treatments by taking deteriorating renal function parameters into account. Implementing the PREVENT risk calculator in the guidelines requires more randomized trials.

Only statins and ezetimibe are currently included in the guidelines for managing dyslipidemia in patients with impaired kidney functions. Due to the rising prevalence of CKD and dyslipidemia in the population, more research should be undertaken in order to allow for more aggressive management of dyslipidemia in this specific population. The decision regarding the management of hyperlipidemia in patients with CKD should be customized for each patient due to the diverse CKD population and multiple stages of CKD

## 10. Conclusions

This comprehensive literature review discusses the intricate relationship between lipid disorders and hemodialysis patients, emphasizing the heightened cardiovascular risk in this population. Despite the well-established benefits of statins in the general population, their efficacy in advanced CKD and dialysis patients remains controversial. Updated and more comprehensive lipid management guidelines for patients with CKD are needed, as the KDIGO guidelines were last revised in 2013, and may have limitations, such as the absence of clear lipid monitoring strategies.

Large-scale trials did not demonstrate a significant reduction in cardiovascular events with statin therapy in ESKD patients on dialysis, which challenges the conventional wisdom regarding the universal applicability of statins in this specific population. Emerging agents like PCSK9 inhibitors, bempedoic acid, and cyclodextrins hold promise for inclusion in future guidelines due to their unique benefits, such as the monthly dosing of PCSK9 inhibitors and the potential to delay CKD progression with cyclodextrins. However, the safety of those agents in CKD and HD patients has only been evaluated in small studies. Larger randomized trials are needed to establish their role in managing dyslipidemia in patients with impaired kidney function. As such, new studies with newer and more effective lipid-lowering agents are crucial to bridge the gap for lipid management in hemodialysis patients who had not been on statin therapy prior to hemodialysis initiation.

In addition to lipid management, the integration of exercise into patient care is an important consideration in optimizing outcomes and enhancing the quality of life for HD patients. Despite limitations in patients with impaired kidney function, such as anemia and deconditioning, physical exercise should be encouraged with the implementation of supervised sessions on inter-dialysis days to mitigate the effect of post-dialysis fatigue syndrome. Given its benefits in reducing the risk of atherosclerotic cardiovascular disease, physical activity should be considered for inclusion in future guidelines for the management of hyperlipidemia in CKD and ESKD patients.

This review underscores the need for tailored lipid management strategies in patients with impaired kidney function, taking into account their unique risk factors, the limitations of current therapies, and the potential of emerging agents. While innovative and new therapies are promising for reducing cardiovascular risk, their high costs and limited accessibility, particularly in resource-poor settings, highlight significant disparities in care. Addressing these challenges requires ongoing research to deepen our understanding of dyslipidemia in this population, alongside strategies that prioritize affordability, and accessibility to improve patient outcomes globally.

## Figures and Tables

**Table 1:** Summary of Major Lipid-Lowering Studies in CKD and ESKD Patients.

Study Name and Year of Publication	Study Population	Intervention	Main Results	Interpretation
4D-2005	A total of 1255 diabetic patients on hemodialysis for less than 2 years; 29% with pre-existing cardiovascular disease.	Atorvastatin 20 mg vs. placebo	Significant reduction in LDL cholesterol (mean reduction of 38%); no significant reduction in major cardiovascular events (HR: 0.94, *p* = 0.51); 14% reduction in combined cardiac events (*p* = 0.03)	Atorvastatin reduced LDL in ESRD patients on dialysis but showed limited benefit in reducing major cardiovascular events; may reduce overall cardiac events.
AURORA-2009	A total of 2776 hemodialysis patients aged between 50 and 80 years old; 40% with cardiovascular disease, and 74% with diabetes.	Rosuvastatin 10 mg vs. placebo	A 43% reduction in LDL cholesterol; 29% reduction in C-reactive protein; no significant reduction in major cardiovascular outcomes	Rosuvastatin effectively lowered LDL and CRP but did not reduce major cardiovascular outcomes in ESRD patients on dialysis.
SHARP-2010	A total of 9270 patients: 6247 with CKD stages 4–5, 3023 on dialysis	Simvastatin 20 mg + Ezetimibe 10 mg vs. simvastatin 20 mg vs. placebo	Significant LDL reduction (by 17% in simvastatin + ezetimibe group, 9% in simvastatin alone); 17% reduction in major atherosclerotic events (*p* = 0.0004) over 4.9 years. A 19% reduction in major atherosclerotic events in CKD patients (*p* = 0.0004) compared to a 13% reduction in major atherosclerotic events in ESKD patients (*p* = 0.02)	Combination therapy with simvastatin and ezetimibe was effective in reducing LDL and atherosclerotic events in advance with more pronounced benefits in CKD patients compared to dialysis patients.

## Data Availability

This study is a literature review and does not include original data. All data supporting the findings are publicly available and can be accessed through the references cited in this manuscript.
